# Assessment of ChatGPT-generated medical Arabic responses for patients with metabolic dysfunction–associated steatotic liver disease

**DOI:** 10.1371/journal.pone.0317929

**Published:** 2025-02-03

**Authors:** Saleh A. Alqahtani, Reem S. AlAhmed, Waleed S. AlOmaim, Saad Alghamdi, Waleed Al-Hamoudi, Khalid Ibrahim Bzeizi, Ali Albenmousa, Alessio Aghemo, Nicola Pugliese, Cesare Hassan, Faisal A. Abaalkhail

**Affiliations:** 1 Liver, Digestive, and Lifestyle Health Research Section, and Organ Transplant Center of Excellence, King Faisal Specialist Hospital and Research Center, Riyadh, Saudi Arabia; 2 Division of Gastroenterology and Hepatology, Weill Cornell Medicine, New York, New York, United States of America; 3 Liver, Digestive, and Lifestyle Health Research Section, and Biostatistics, Epidemiology and Scientific Computing Department, King Faisal Specialist Hospital and Research Center, Riyadh, Saudi Arabia; 4 Department of Pathology and Laboratory Medicine, King Faisal Specialist Hospital and Research Center, Riyadh, Saudi Arabia; 5 Organ Transplant Center of Excellence, King Faisal Specialist Hospital and Research Center, Riyadh, Saudi Arabia; 6 Department of Biomedical Sciences, Humanitas University, Pieve Emanuele (MI), Italy; 7 Division of Internal Medicine and Hepatology, Department of Gastroenterology, IRCCS Humanitas Research Hospital, Rozzano (MI), Italy; 8 Gastroenterology Section, Department of Medicine, King Faisal Specialist Hospital and Research Center, Riyadh, Saudi Arabia; 9 College of Medicine, Alfaisal University, Riyadh, Saudi Arabia; Universita degli Studi della Campania Luigi Vanvitelli Scuola di Medicina e Chirurgia, ITALY

## Abstract

**Background and aim:**

Artificial intelligence (AI)-powered chatbots, such as Chat Generative Pretrained Transformer (ChatGPT), have shown promising results in healthcare settings. These tools can help patients obtain real-time responses to queries, ensuring immediate access to relevant information. The study aimed to explore the potential use of ChatGPT-generated medical Arabic responses for patients with metabolic dysfunction–associated steatotic liver disease (MASLD).

**Methods:**

An English patient questionnaire on MASLD was translated to Arabic. The Arabic questions were then entered into ChatGPT 3.5 on November 12, 2023. The responses were evaluated for accuracy, completeness, and comprehensibility by 10 Saudi MASLD experts who were native Arabic speakers. Likert scales were used to evaluate: 1) Accuracy, 2) Completeness, and 3) Comprehensibility. The questions were grouped into 3 domains: (1) Specialist referral, (2) Lifestyle, and (3) Physical activity.

**Results:**

Accuracy mean score was 4.9 ± 0.94 on a 6-point Likert scale corresponding to “Nearly all correct.” Kendall’s coefficient of concordance (KCC) ranged from 0.025 to 0.649, with a mean of 0.28, indicating moderate agreement between all 10 experts. Mean completeness score was 2.4 ± 0.53 on a 3-point Likert scale corresponding to “Comprehensive” (KCC: 0.03–0.553; mean: 0.22). Comprehensibility mean score was 2.74 ± 0.52 on a 3-point Likert scale, which indicates the responses were “Easy to understand” (KCC: 0.00–0.447; mean: 0.25).

**Conclusion:**

MASLD experts found that ChatGPT responses were accurate, complete, and comprehensible. The results support the increasing trend of leveraging the power of AI chatbots to revolutionize the dissemination of information for patients with MASLD. However, many AI-powered chatbots require further enhancement of scientific content to avoid the risks of circulating medical misinformation.

## Introduction

Metabolic dysfunction–associated steatotic liver disease (MASLD), formerly known as non-alcoholic fatty liver disease (NAFLD), is a global health concern, closely linked to the obesity epidemic and sedentary lifestyles [1, 2]. MASLD involves a full spectrum of conditions resulting from metabolic imbalances, such as metabolic dysfunction-associated steatohepatitis (MASH), previously called non-alcoholic steatohepatitis (NASH) [1, 3]. MASLD and MASH pose enormous financial and health burdens across countries, including those in the Arabic-speaking world [4–6]. Early detection and treatment of MASLD are crucial to prevent the progression of more severe stages like cirrhosis and hepatocellular carcinoma [1, 7]. However, barriers to healthcare access and patient literacy may create challenges in managing this condition effectively [8, 9].

In the digital age, artificial intelligence (AI) applications in healthcare offer innovative solutions to such challenges. Chatbots powered by advanced AI models, like the Chat Generative Pretrained Transformer (ChatGPT), can supplement patient education and engagement outside the clinical setting [10]. With their ability to process and produce human-like text, these AI tools can deliver instant, reliable medical information and support, potentially transforming patient self-management practices [11, 12].

From a previous study aimed to determine ChatGPT’s effectiveness in answering patient inquiries concerning MASLD and associated lifestyle factors, findings indicated that ChatGPT delivered accurate (mean score of 4.84 on a 6-point Likert scale), comprehensive (mean score of 2.08 on a 3-point scale), and easy to understand (mean score of 2.87 on a 3-point scale) responses. Nonetheless, it is noteworthy that the variability in ChatGPT’s responses may be attributed to factors such as the training dataset, context, and language [13].

Despite the promise of AI-powered interventions, their effectiveness for Arabic-speaking patients with MASLD remains underexplored. We aimed to explore the potential use of ChatGPT in generating medical responses in Arabic for patients with MASLD, assessing its accuracy, reliability, and comprehensiveness as an informative resource.

## Materials and methods

A cross-sectional study assessed the effectiveness of ChatGPT in providing medical responses to Arabic-speaking patients with MASLD. The process followed three main steps: 1) A validated English-language patient questionnaire on MASLD [13], was translated into Arabic by the MASLD experts and an independent researcher, ensuring linguistic and contextual accuracy from a patient perspective; 2) The translated questions were then entered separately into ChatGPT 3.5 on November 12, 2023, simulating a realistic scenario where a patient seeks information regarding MASLD; and 3) Ten MASLD experts from Saudi Arabia, who were native Arabic speakers and fluent in English, independently evaluated the AI-generated responses. The data was collected from 01/31/2024 through 02/10/2024. For the survey and questionnaire, we primarily used Classical Arabic, which is the standard for formal and business writing, ensuring a common linguistic framework across diverse Arabic-speaking populations.

Three domains were assessed using respective Likert scales: 1) Accuracy: Responses were rated on a 6-point Likert scale ranging from ’Completely incorrect’ to ’Correct’; 2) Completeness: A 3-point Likert scale was utilized, categorizing responses as ’Incomplete’, ’Adequate’, or ’Comprehensive’; and 3) Comprehensibility: The intelligibility of responses was determined using a 3-point Likert scale marked by ’Difficult’, ’Partly difficult’, and ’Easy to understand’.

An additional open-ended question was integrated into the Arabic questionnaire to gather detailed feedback and expert commentary on the AI-generated response quality. This structured evaluation method aimed to capture the nuanced perspectives of clinical experts regarding the application of ChatGPT in patient education and its potential role in improving MASLD patient care within Arabic-speaking populations.

### Statistical analysis

The data was analyzed using the Statistical Package for Social Sciences (SPSS), version 28 (IBM Corp., N.Y., USA). To assess the potential usability of ChatGPT’s Arabic responses for patients with MASLD, the non-parametric Kendall Tau’s correlation test was employed. It examined the association between experts’ ratings, using ordinal data from Likert scale assessments for the three domains, to determine the direction and strength of relationships between the variables under study. The mean scores, measured on 6- and 3-point Likert scales, Kendall’s coefficient of concordance, and range values were expressed.

### Ethical statement

Ethical approval for this study was obtained from the Research Ethics Committee (REC) of King Faisal Specialist Hospital & Research Center, Riyadh, Saudi Arabia (RAC #2241013) on 01/29/2024. The REC recommended the approval of the study with a waiver of signing and documentation of consent. The decision of participant MASLD experts to submit the survey was considered consent.

## Results

### Accuracy

**Fig 1:**
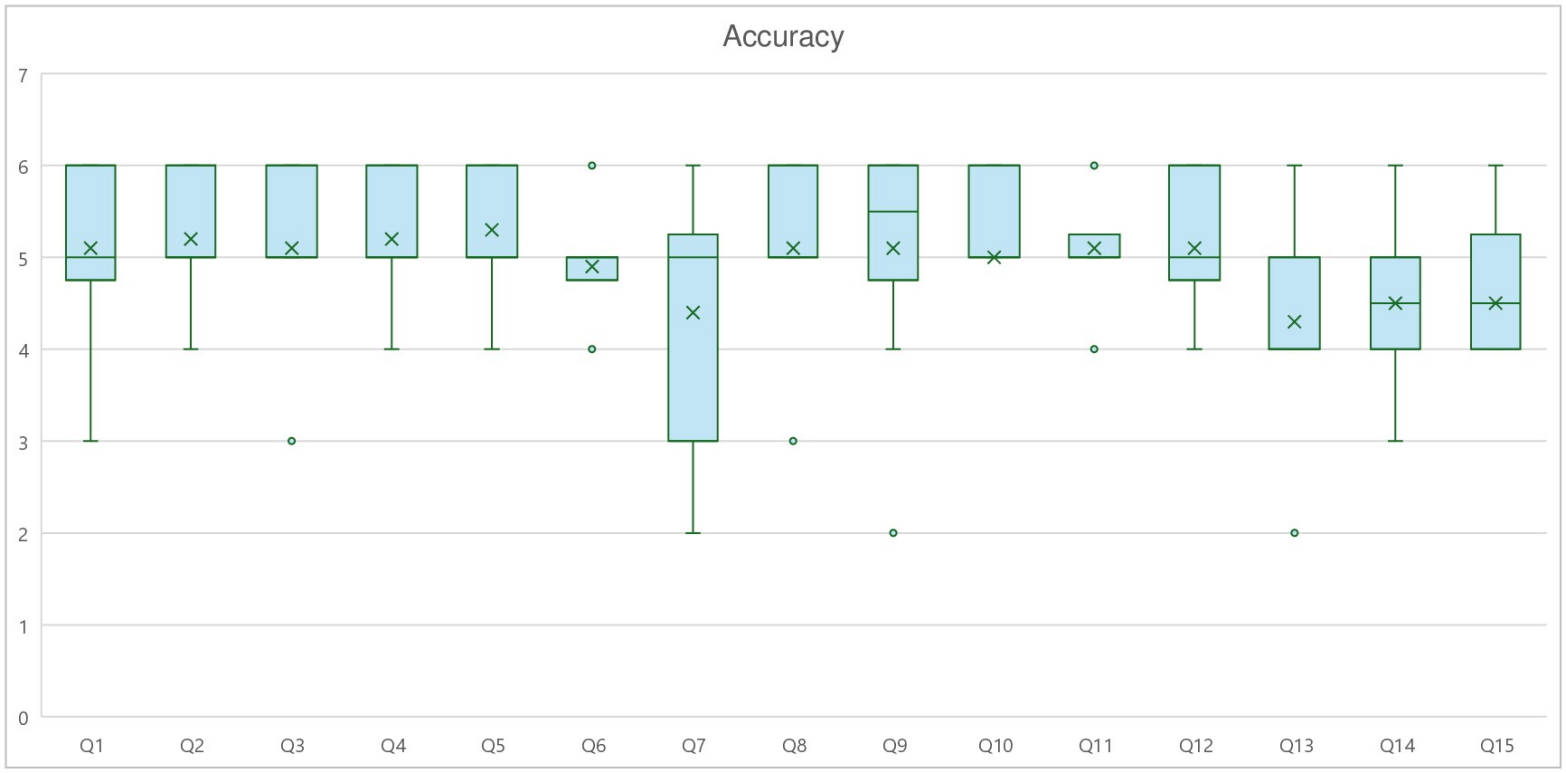
Accuracy score. Box plot showing the distribution of accuracy scores for each question. Graph shows the interquartile range (box), median (horizontal line), mean (dot), and outliers (whiskers).

The mean score for accuracy was 4.92 ± 0.94 on a 6-point Likert scale corresponding to “Nearly all correct”. Kendall’s coefficient of concordance ranged from 0.025 to 0.649, with a mean of 0.28, indicating a moderate level of agreement among all 10 experts. The highest mean square was for question 5, with a mean of 5.3 corresponding to “Correct”. The lowest mean was question 13, with a mean score of 4.3, corresponding to “More correct than incorrect”. Among the three domains, Physical Activity had the highest accuracy mean of 5.07 ± 0.83, while specialist referral had the lowest mean score of 4.70 ± 1.02 (Fig 1).

### Completeness

**Fig 2:**
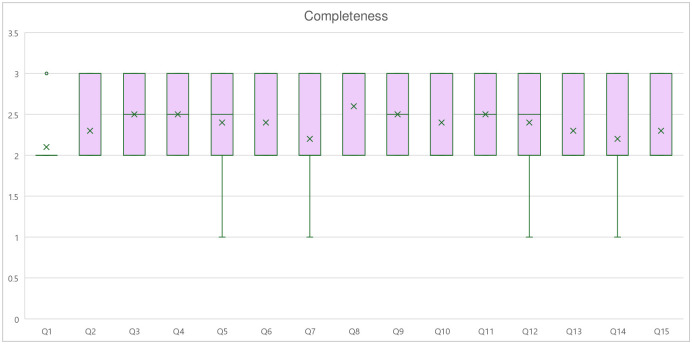
Completeness score. Box plot showing the distribution of completeness scores for each question. Graph shows the interquartile range (box), median (horizontal line), mean (dot), and outliers (whiskers).

The mean score for completeness was 2.37 ± 0.53 on a 3-point Likert scale, corresponding to “Comprehensive”. Kendall’s coefficient ranged from 0.03 to 0.553, with a mean of 0.22, indicating a moderate level of agreement among all 10 experts. The highest question mean score was Q8 of 2.6, corresponding to “Comprehensive”. The lowest mean was question 1, with a mean score of 2.1, corresponding to “Adequate”. Among the three domains, Physical Activity had the highest mean score of 2.43 ± 0.57, while specialist referral had the lowest mean score of 2.20 ± 0.48 (Fig 2).

### Comprehensibility

**Fig 3:**
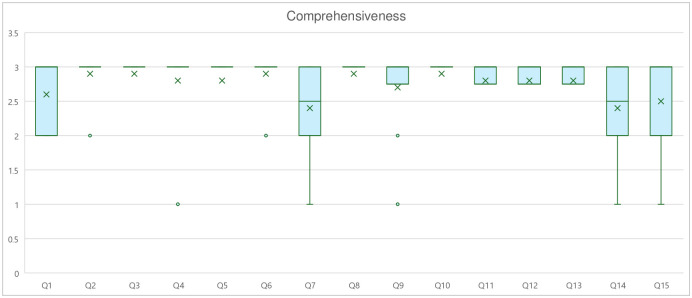
Comprehensibility score. Box plot showing the distribution of comprehensibility scores for each question. Graph shows the interquartile range (box), median (horizontal line), mean (dot), and outliers (whiskers).

The average comprehensibility score was 2.74 ± 0.52, which indicates that the ChatGPT-generated responses were “Easy to understand”. Kendall’s coefficient ranged from 0.00 to 0.447, with a mean of 0.25, indicating a moderate level of agreement among all 10 experts. The highest question mean score of 2.9 was questions 2, 3, 6, 8, and 10. The lowest question mean score of 2.4 was questions 7 and 14. Among the three domains, Physical Activity had the highest mean score of 2.83 ± 0.38, while specialist referral had the lowest mean score of 2.50 ± 0.63 (Fig 3).

### Expert comments

When comparing responses with the highest/lowest frequency of the expert comments, the following questions generated responses with no comments by the expert (Questions 8–10 and 12). The highest questions that had more than one expert comment were questions 1, 5, and 14. Grouping comments by theme, the following had been identified to be the most repeated comments among the experts: 1) The generated responses used the term “NAFLD/NASH” instead of “MASLD/MASH”; 2) The Arabic-generated response translation of “Biopsy”; 3) The Arabic-generated response translation of “MRI”; and 4) The Arabic-generated response sentences on alcohol consumption.

## Discussion

AI is significantly impacting the medical field, including gastroenterology and hepatology [14, 15]. In recent years, AI has been successfully applied in liver pathology and radiology to improve diagnostic accuracy and reduce inter- and intra-observer variability [14–16]. Recently, significant attention has been paid to the clinical applications of AI-based chatbots, specifically ChatGPT in various contexts, including its potential use as an immediate, free, and on-demand information dissemination tool for patients with MASLD [14]. Identifying effective information dissemination tools for patients with MASLD is a clinical priority for disease management, as MASLD management needs a multidisciplinary approach [17]. Patient education and information dissemination are an essential component for helping patients in achieving and maintaining lifestyle changes [18, 19]. AI-based chatbots could be a valuable tool for patients by providing simplified explanations and guidance on first-line treatment options and disease management such as weight loss and physical activity recommendations.

Pugliese et al. [13] recently conducted the first study on ChatGPT 3.5 as an information dissemination tool for patients with MASLD, demonstrating that ChatGPT 3.5 can provide understandable and complete answers from the patient’s perspective to 15 pre-defined MASLD-related questions in English. The AI-generated answers were evaluated by 10 experts and found to be relatively accurate [13]. In addition, preliminary data from another study by the same authors showed that using a different language from English did not seem to affect the effectiveness of ChatGPT as a resource tool for patients with MASLD [20]. To date, no study assessed the effectiveness of AI-powered interventions for Arabic-speaking patients with MASLD.

In our study, we involved 10 MASLD experts from Saudi Arabia who were native Arabic speakers and evaluated the same set of questions that were previously analyzed in English. We found that ChatGPT’s ability to advise patients with MASLD was not affected by language, as the Arabic answers were deemed to be complete (with a mean score of 2.4 on a 3-point scale) and comprehensible (with a mean score of 2.74 on a 3-point scale). However, consistent with other studies, the accuracy of ChatGPT still requires improvement, with a mean score of 4.9 on a 6-point Likert scale (Table 1). So, while the Arabic language does not influence the completeness and accuracy of ChatGPT generated answers, it also does not improve the inaccuracies observed in clinically meaningful answers. Similar to a previous study conducted in the English language [13], the Physical Activity domain had the highest score as well for the Arabic questionnaire (Table 2).

**Table 1:** Comparing the mean score result between the Arabic and English responses [13].

Evaluation Parameters	Arabic	English
**Accuracy**	4.92 ± 0.94	4.84 ± 0.74
**Completeness**	2.37 ± 0.53	2.08 ± 0.3
**comprehensibility**	2.74 ± 0.52	2.87 ± 0.14

**Table 2:** Comparing domains mean score result between the Arabic and English responses [13].

**Accuracy Mean Score**	**Arabic**	**English**
Highest domain	**Physical Activity** 5.07 ± 0.83	**Physical Activity** 5.56 ± 0.56
Lowest domain	**Specialist Referral** 4.70 ± 1.02	**Specialist Referral** 3.9 ± 1.44
**Completeness Mean Score**	**Arabic**	**English**
Highest domain	**Physical Activity** 2.43 ± 0.57	**Physical Activity** 2.46 ± 0.5
Lowest domain	**Specialist Referral** 2.20 ± 0.48	**Specialist Referral** 1.73 ± 0.82

### Limitations

Ten experts in the field of MASLD conducted the ratings using Likert scales. However, it is important to note that such scales have limitations as they allow for partial accuracy ratings. This is unacceptable in the medical field as it can lead to misunderstandings and dangerous consequences for patients. Another limitation is the availability of new and potentially better versions of ChatGPT (ChatGPT 4), as the study used version 3.5. However, it should be noted that ChatGPT 4 is not freely accessible to patients and thus it is unlikely to be used any time soon. While a variety of large language models are accessible, including free options, our decision to employ ChatGPT was primarily driven by methodological consistency. To ensure a reliable comparison between English and Arabic responses, it was crucial to maintain a standardized approach. By utilizing the same AI tool, we could isolate the impact of language differences on the generated content. We acknowledge the rapid advancements in AI technology and the potential benefits of exploring diverse models which may improve in accuracy and cultural relevance. Future research endeavors will undoubtedly involve a comparative analysis of various AI tools to assess their relative strengths and weaknesses in different language contexts.

In addition, it is crucial to consider the impact of socio-cultural factors on ChatGPT responses. The sociocultural background of the patient determines the tool’s capacity to offer culturally sensitive guidance, and patient preferences, health literacy levels, and cultural quirks may all affect how successful the responses are. Therefore, even if ChatGPT is a useful tool, its use needs to be done with consideration for the patients’ cultural variety [20] Chatbots also have other known limitations, including the risk of generating content that may not be grounded in evidence-based knowledge, a phenomenon known as ’hallucinations’ [21]. Retrieval augmented generation (RAG) is a potential method to address this issue. RAG combines the response-generating ability of AI-based chatbots with the ability to pull in verified information from external sources, resulting in more accurate and complete answers. There is a growing trend not only in acquiring information from AI-based apps and services but also in decision-making based on such information. Hence, the professional community should use AI responsibly by following the principles and ethics associated with it.

### Conclusions

This study addresses the critical requirement for AI tools in the Arabic-speaking world, where the prevalence of MASLD is estimated to be higher than in Western countries [22]. Although our study confirms the promising results obtained by previous studies, the universal adoption of ChatGPT as a resource tool for MASLD patients is challenging [13, 20]. The identified limitations highlight the need for continued improvement of AI models in healthcare settings. Such improvement requires collaboration between AI experts and healthcare professionals, which is necessary and crucial. While the study results showcase that the AI-generated responses are accurate and consistent, patients should be informed not to replace conventional doctor visits with these technologies, as they facilitate educational patient material specifically, and are not a way to have a medical diagnosis or consultation.

## Supporting information

S1 Table Accuracy Likert scale reference. (DOCX)

S2 Table Completeness Likert scale reference. (DOCX)

S3 Table Comprehensiveness Likert scale reference. (DOCX)

S4 Table Accuracy coded responses. (DOCX)

S5 Table Completeness coded responses. (DOCX)

S6 Table Completeness coded responses. (DOCX)

S7 Table Accuracy—Kendall’s tau analysis. (DOCX)

S8 Table Completeness Kendall’s tau. (DOCX)

S9 Table Comprehensiveness Kendall’s tau. (DOCX)

S10 Table Arabic questionnaire. (DOCX)

S11 Table ChatGPT generated responses. (DOCX)
