# Supplementary material for: Assessment of ChatGPT-generated medical Arabic responses for patients with metabolic dysfunction–associated steatotic liver disease
Source: PLoS One. 2025 Feb 3;20(2):e0317929. doi: 10.1371/journal.pone.0317929 (PMC11790096; doi:10.1371/journal.pone.0317929)
Supplement: S1 Table — (DOCX) [file pone.0317929.s001.docx]

**S1 Table. Accuracy Likert Scale Reference**

| **Response Options** | **Code** | **Range** |
| --- | --- | --- |
| Correct | 6 | 5.26 - 6 |
| Nearly all correct | 5 | 4.43 - 5.16 |
| More correct than incorrect | 4 | 3.60 - 4.33 |
| Approximately equally correct and incorrect | 3 | 2.76 - 3.50 |
| More incorrect than correct | 2 | 1.93 - 2.66 |
| Completely incorrect | 1 | 1.00 - 1.83 |
