# Supplementary material for: Assessment of ChatGPT-generated medical Arabic responses for patients with metabolic dysfunction–associated steatotic liver disease
Source: PLoS One. 2025 Feb 3;20(2):e0317929. doi: 10.1371/journal.pone.0317929 (PMC11790096; doi:10.1371/journal.pone.0317929)
Supplement: S2 Table — (DOCX) [file pone.0317929.s002.docx]

**S2 Table. Completeness Likert Scale Reference**

| **Question** | **Code** | | **Range** |
| --- | --- | --- | --- |
| Incomplete | | 1 | 1-1.66 |
| Adequate | | 2 | 1.76 - 2.32 |
| Comprehensive | | 3 | 2.43 - 3.00 |
