# Supplementary material for: Assessment of ChatGPT-generated medical Arabic responses for patients with metabolic dysfunction–associated steatotic liver disease
Source: PLoS One. 2025 Feb 3;20(2):e0317929. doi: 10.1371/journal.pone.0317929 (PMC11790096; doi:10.1371/journal.pone.0317929)
Supplement: S4 Table — (DOCX) [file pone.0317929.s004.docx]

**S4 Table. Accuracy Coded responses**

| respondent_id | Q1_1 | Q2_1 | Q3_1 | Q4_1 | Q5_1 | Q6_1 | Q7_1 | Q8_1 | Q9_1 | Q10_1 | Q11_1 | Q12_1 | Q13_1 | Q14_1 | Q15_1 |
| --- | --- | --- | --- | --- | --- | --- | --- | --- | --- | --- | --- | --- | --- | --- | --- |
| RI_1 | 3 | 5 | 5 | 5 | 5 | 5 | 5 | 5 | 5 | 5 | 5 | 5 | 5 | 4 | 4 |
| RI_2 | 4 | 5 | 5 | 5 | 5 | 5 | 3 | 5 | 4 | 5 | 5 | 5 | 4 | 4 | 4 |
| RI_3 | 6 | 4 | 3 | 4 | 4 | 4 | 3 | 3 | 2 | 2 | 5 | 4 | 2 | 4 | 2 |
| RI_4 | 5 | 5 | 5 | 6 | 6 | 5 | 4 | 6 | 6 | 5 | 6 | 6 | 4 | 5 | 6 |
| RI_5 | 5 | 6 | 6 | 5 | 6 | 5 | 6 | 5 | 6 | 6 | 5 | 4 | 4 | 6 | 6 |
| RI_6 | 5 | 5 | 6 | 6 | 6 | 4 | 5 | 5 | 6 | 5 | 5 | 5 | 5 | 5 | 5 |
| RI_7 | 5 | 5 | 5 | 5 | 5 | 5 | 5 | 5 | 5 | 5 | 5 | 5 | 5 | 5 | 5 |
| RI_8 | 6 | 6 | 5 | 5 | 6 | 5 | 5 | 6 | 6 | 6 | 5 | 5 | 4 | 5 | 5 |
| RI_9 | 6 | 5 | 5 | 5 | 5 | 5 | 2 | 5 | 5 | 5 | 4 | 6 | 4 | 4 | 4 |
| RI_10 | 6 | 6 | 6 | 6 | 5 | 6 | 6 | 6 | 6 | 6 | 6 | 6 | 6 | 3 | 4 |
|  |  |  |  |  |  |  |  |  |  |  |  |  |  |  |  |
| sum | 51 | 52 | 51 | 52 | 53 | 49 | 44 | 51 | 51 | 50 | 51 | 51 | 43 | 45 | 45 |
| Mean | 5.1 | 5.2 | 5.1 | 5.2 | 5.3 | 4.9 | 4.4 | 5.1 | 5.1 | 5 | 5.1 | 5.1 | 4.3 | 4.5 | 4.5 |
| SD | 0.994428926 | 0.632455532 | 0.875595036 | 0.632455532 | 0.674948558 | 0.567646212 | 1.349897115 | 0.875595036 | 1.286683938 | 1.154700538 | 0.567646212 | 0.737864787 | 1.059349905 | 0.849836586 | 1.178511302 |
