# Supplementary material for: Assessment of ChatGPT-generated medical Arabic responses for patients with metabolic dysfunction–associated steatotic liver disease
Source: PLoS One. 2025 Feb 3;20(2):e0317929. doi: 10.1371/journal.pone.0317929 (PMC11790096; doi:10.1371/journal.pone.0317929)
Supplement: S5 Table — (DOCX) [file pone.0317929.s005.docx]

**S5 Table. Completeness Coded responses**

| respondent_id | Q1_2 | Q2_2 | Q3_2 | Q4_2 | Q5_2 | Q6_2 | Q7_2 | Q8_2 | Q9_2 | Q10_2 | Q11_2 | Q12_2 | Q13_2 | Q14_2 | Q15_2 |
| --- | --- | --- | --- | --- | --- | --- | --- | --- | --- | --- | --- | --- | --- | --- | --- |
| RI_1 | 2 | 3 | 3 | 3 | 3 | 3 | 3 | 3 | 3 | 3 | 3 | 3 | 3 | 3 | 3 |
| RI_2 | 2 | 3 | 3 | 3 | 3 | 3 | 2 | 3 | 2 | 2 | 3 | 2 | 2 | 2 | 3 |
| RI_3 | 2 | 2 | 2 | 2 | 1 | 2 | 2 | 2 | 2 | 2 | 2 | 1 | 2 | 2 | 2 |
| RI_4 | 2 | 2 | 2 | 2 | 2 | 2 | 2 | 3 | 3 | 2 | 3 | 3 | 2 | 2 | 2 |
| RI_5 | 2 | 2 | 2 | 2 | 3 | 3 | 3 | 2 | 2 | 2 | 2 | 2 | 3 | 3 | 3 |
| RI_6 | 2 | 2 | 3 | 2 | 3 | 2 | 2 | 2 | 2 | 2 | 2 | 3 | 2 | 2 | 2 |
| RI_7 | 2 | 2 | 2 | 2 | 2 | 2 | 2 | 2 | 2 | 2 | 2 | 2 | 2 | 2 | 2 |
| RI_8 | 2 | 2 | 2 | 3 | 3 | 2 | 2 | 3 | 3 | 3 | 2 | 2 | 2 | 3 | 2 |
| RI_9 | 2 | 2 | 3 | 3 | 2 | 2 | 1 | 3 | 3 | 3 | 3 | 3 | 2 | 2 | 2 |
| RI_10 | 3 | 3 | 3 | 3 | 2 | 3 | 3 | 3 | 3 | 3 | 3 | 3 | 3 | 1 | 2 |
|  |  |  |  |  |  |  |  |  |  |  |  |  |  |  |  |
| sum | 21 | 23 | 25 | 25 | 24 | 24 | 22 | 26 | 25 | 24 | 25 | 24 | 23 | 22 | 23 |
| Mean | 2.1 | 2.3 | 2.5 | 2.5 | 2.4 | 2.4 | 2.2 | 2.6 | 2.5 | 2.4 | 2.5 | 2.4 | 2.3 | 2.2 | 2.3 |
| SD | 0.316227766 | 0.483045892 | 0.527046277 | 0.527046277 | 0.699205899 | 0.516397779 | 0.632455532 | 0.516397779 | 0.527046277 | 0.516397779 | 0.527046277 | 0.699205899 | 0.483045892 | 0.632455532 | 0.483045892 |
