# Supplementary material for: Assessment of ChatGPT-generated medical Arabic responses for patients with metabolic dysfunction–associated steatotic liver disease
Source: PLoS One. 2025 Feb 3;20(2):e0317929. doi: 10.1371/journal.pone.0317929 (PMC11790096; doi:10.1371/journal.pone.0317929)
Supplement: S6 Table — (DOCX) [file pone.0317929.s006.docx]

**S6 Table. Completeness Coded responses**

| respondent_id | Q1_3 | Q2_3 | Q3_3 | Q4_3 | Q5_3 | Q6_3 | Q7_3 | Q8_3 | Q9_3 | Q10_3 | Q11_3 | Q12_3 | Q13_3 | Q14_3 | Q15_3 |
| --- | --- | --- | --- | --- | --- | --- | --- | --- | --- | --- | --- | --- | --- | --- | --- |
| RI_1 | 3 | 3 | 3 | 3 | 3 | 3 | 3 | 3 | 3 | 3 | 3 | 3 | 3 | 3 | 3 |
| RI_2 | 2 | 3 | 3 | 3 | 3 | 3 | 2 | 3 | 2 | 3 | 3 | 2 | 2 | 1 | 2 |
| RI_3 | 2 | 2 | 2 | 1 | 1 | 2 | 1 | 2 | 1 | 2 | 2 | 2 | 2 | 2 | 2 |
| RI_4 | 3 | 3 | 3 | 3 | 3 | 3 | 2 | 3 | 3 | 3 | 3 | 3 | 3 | 3 | 3 |
| RI_5 | 2 | 3 | 3 | 3 | 3 | 3 | 3 | 3 | 3 | 3 | 3 | 3 | 3 | 3 | 3 |
| RI_6 | 3 | 3 | 3 | 3 | 3 | 3 | 3 | 3 | 3 | 3 | 3 | 3 | 3 | 3 | 3 |
| RI_7 | 3 | 3 | 3 | 3 | 3 | 3 | 3 | 3 | 3 | 3 | 3 | 3 | 3 | 3 | 3 |
| RI_8 | 3 | 3 | 3 | 3 | 3 | 3 | 2 | 3 | 3 | 3 | 2 | 3 | 3 | 2 | 3 |
| RI_9 | 2 | 3 | 3 | 3 | 3 | 3 | 2 | 3 | 3 | 3 | 3 | 3 | 3 | 2 | 2 |
| RI_10 | 3 | 3 | 3 | 3 | 3 | 3 | 3 | 3 | 3 | 3 | 3 | 3 | 3 | 2 | 1 |
|  |  |  |  |  |  |  |  |  |  |  |  |  |  |  |  |
| sum | 26 | 29 | 29 | 28 | 28 | 29 | 24 | 29 | 27 | 29 | 28 | 28 | 28 | 24 | 25 |
| Mean | 2.6 | 2.9 | 2.9 | 2.8 | 2.8 | 2.9 | 2.4 | 2.9 | 2.7 | 2.9 | 2.8 | 2.8 | 2.8 | 2.4 | 2.5 |
| SD | 0.516397779 | 0.316227766 | 0.316227766 | 0.632455532 | 0.632455532 | 0.316227766 | 0.699205899 | 0.316227766 | 0.674948558 | 0.316227766 | 0.421637021 | 0.421637021 | 0.421637021 | 0.699205899 | 0.707106781 |
