# Supplementary material for: Assessment of ChatGPT-generated medical Arabic responses for patients with metabolic dysfunction–associated steatotic liver disease
Source: PLoS One. 2025 Feb 3;20(2):e0317929. doi: 10.1371/journal.pone.0317929 (PMC11790096; doi:10.1371/journal.pone.0317929)
Supplement: S7 Table — (DOCX) [file pone.0317929.s007.docx]

**S7 Table. Accuracy - Kendall's tau Analysis**

|  | | | respondent_id |
| --- | --- | --- | --- |
| Kendall's tau_b | respondent_id | Correlation Coefficient | 1.000 |
|  |  | Sig. (2-tailed) | . |
|  |  | N | 10 |
|  | Q1_1 | Correlation Coefficient | .649^*^ |
|  |  | Sig. (2-tailed) | .016 |
|  |  | N | 10 |
|  | Q2_1 | Correlation Coefficient | .430 |
|  |  | Sig. (2-tailed) | .120 |
|  |  | N | 10 |
|  | Q3_1 | Correlation Coefficient | .316 |
|  |  | Sig. (2-tailed) | .254 |
|  |  | N | 10 |
|  | Q4_1 | Correlation Coefficient | .258 |
|  |  | Sig. (2-tailed) | .351 |
|  |  | N | 10 |
|  | Q5_1 | Correlation Coefficient | .083 |
|  |  | Sig. (2-tailed) | .764 |
|  |  | N | 10 |
|  | Q6_1 | Correlation Coefficient | .342 |
|  |  | Sig. (2-tailed) | .218 |
|  |  | N | 10 |
|  | Q7_1 | Correlation Coefficient | .221 |
|  |  | Sig. (2-tailed) | .400 |
|  |  | N | 10 |
|  | Q8_1 | Correlation Coefficient | .373 |
|  |  | Sig. (2-tailed) | .178 |
|  |  | N | 10 |
|  | Q9_1 | Correlation Coefficient | .316 |
|  |  | Sig. (2-tailed) | .241 |
|  |  | N | 10 |
|  | Q10_1 | Correlation Coefficient | .430 |
|  |  | Sig. (2-tailed) | .120 |
|  |  | N | 10 |
|  | Q11_1 | Correlation Coefficient | -.031 |
|  |  | Sig. (2-tailed) | .911 |
|  |  | N | 10 |
|  | Q12_1 | Correlation Coefficient | .402 |
|  |  | Sig. (2-tailed) | .141 |
|  |  | N | 10 |
|  | Q13_1 | Correlation Coefficient | .264 |
|  |  | Sig. (2-tailed) | .328 |
|  |  | N | 10 |
|  | Q14_1 | Correlation Coefficient | -.026 |
|  |  | Sig. (2-tailed) | .923 |
|  |  | N | 10 |
|  | Q15_1 | Correlation Coefficient | -.025 |
|  |  | Sig. (2-tailed) | .925 |
|  |  | N | 10 |
