# Supplementary material for: Assessment of ChatGPT-generated medical Arabic responses for patients with metabolic dysfunction–associated steatotic liver disease
Source: PLoS One. 2025 Feb 3;20(2):e0317929. doi: 10.1371/journal.pone.0317929 (PMC11790096; doi:10.1371/journal.pone.0317929)
Supplement: S8 Table — (DOCX) [file pone.0317929.s008.docx]

**S8 Table. Completeness Kendall's tau**

|  | | | respondent_id |
| --- | --- | --- | --- |
| Kendall's tau_b | respondent_id | Correlation Coefficient | 1.000 |
|  |  | Sig. (2-tailed) | . |
|  |  | N | 10 |
|  | Q1_2 | Correlation Coefficient | .447 |
|  |  | Sig. (2-tailed) | .117 |
|  |  | N | 10 |
|  | Q2_2 | Correlation Coefficient | -.228 |
|  |  | Sig. (2-tailed) | .425 |
|  |  | N | 10 |
|  | Q3_2 | Correlation Coefficient | .030 |
|  |  | Sig. (2-tailed) | .917 |
|  |  | N | 10 |
|  | Q4_2 | Correlation Coefficient | .149 |
|  |  | Sig. (2-tailed) | .602 |
|  |  | N | 10 |
|  | Q5_2 | Correlation Coefficient | -.194 |
|  |  | Sig. (2-tailed) | .483 |
|  |  | N | 10 |
|  | Q6_2 | Correlation Coefficient | -.243 |
|  |  | Sig. (2-tailed) | .394 |
|  |  | N | 10 |
|  | Q7_2 | Correlation Coefficient | -.201 |
|  |  | Sig. (2-tailed) | .468 |
|  |  | N | 10 |
|  | Q8_2 | Correlation Coefficient | .061 |
|  |  | Sig. (2-tailed) | .831 |
|  |  | N | 10 |
|  | Q9_2 | Correlation Coefficient | .268 |
|  |  | Sig. (2-tailed) | .347 |
|  |  | N | 10 |
|  | Q10_2 | Correlation Coefficient | .365 |
|  |  | Sig. (2-tailed) | .201 |
|  |  | N | 10 |
|  | Q11_2 | Correlation Coefficient | -.089 |
|  |  | Sig. (2-tailed) | .754 |
|  |  | N | 10 |
|  | Q12_2 | Correlation Coefficient | .194 |
|  |  | Sig. (2-tailed) | .483 |
|  |  | N | 10 |
|  | Q13_2 | Correlation Coefficient | -.033 |
|  |  | Sig. (2-tailed) | .909 |
|  |  | N | 10 |
|  | Q14_2 | Correlation Coefficient | -.316 |
|  |  | Sig. (2-tailed) | .254 |
|  |  | N | 10 |
|  | Q15_2 | Correlation Coefficient | -.553 |
|  |  | Sig. (2-tailed) | .053 |
|  |  | N | 10 |
