# Supplementary material for: Assessment of ChatGPT-generated medical Arabic responses for patients with metabolic dysfunction–associated steatotic liver disease
Source: PLoS One. 2025 Feb 3;20(2):e0317929. doi: 10.1371/journal.pone.0317929 (PMC11790096; doi:10.1371/journal.pone.0317929)
Supplement: S9 Table — (DOCX) [file pone.0317929.s009.docx]

**S9 Table. Comprehensiveness Kendall's tau**

|  | | | respondent_id |
| --- | --- | --- | --- |
| Kendall's tau_b | respondent_id | Correlation Coefficient | 1.000 |
|  |  | Sig. (2-tailed) | . |
|  |  | N | 10 |
|  | Q1_3 | Correlation Coefficient | .183 |
|  |  | Sig. (2-tailed) | .522 |
|  |  | N | 10 |
|  | Q2_3 | Correlation Coefficient | .248 |
|  |  | Sig. (2-tailed) | .384 |
|  |  | N | 10 |
|  | Q3_3 | Correlation Coefficient | .248 |
|  |  | Sig. (2-tailed) | .384 |
|  |  | N | 10 |
|  | Q4_3 | Correlation Coefficient | .248 |
|  |  | Sig. (2-tailed) | .384 |
|  |  | N | 10 |
|  | Q5_3 | Correlation Coefficient | .248 |
|  |  | Sig. (2-tailed) | .384 |
|  |  | N | 10 |
|  | Q6_3 | Correlation Coefficient | .248 |
|  |  | Sig. (2-tailed) | .384 |
|  |  | N | 10 |
|  | Q7_3 | Correlation Coefficient | .138 |
|  |  | Sig. (2-tailed) | .616 |
|  |  | N | 10 |
|  | Q8_3 | Correlation Coefficient | .248 |
|  |  | Sig. (2-tailed) | .384 |
|  |  | N | 10 |
|  | Q9_3 | Correlation Coefficient | .398 |
|  |  | Sig. (2-tailed) | .154 |
|  |  | N | 10 |
|  | Q10_3 | Correlation Coefficient | .248 |
|  |  | Sig. (2-tailed) | .384 |
|  |  | N | 10 |
|  | Q11_3 | Correlation Coefficient | .000 |
|  |  | Sig. (2-tailed) | 1.000 |
|  |  | N | 10 |
|  | Q12_3 | Correlation Coefficient | .447 |
|  |  | Sig. (2-tailed) | .117 |
|  |  | N | 10 |
|  | Q13_3 | Correlation Coefficient | .447 |
|  |  | Sig. (2-tailed) | .117 |
|  |  | N | 10 |
|  | Q14_3 | Correlation Coefficient | -.138 |
|  |  | Sig. (2-tailed) | .616 |
|  |  | N | 10 |
|  | Q15_3 | Correlation Coefficient | -.201 |
|  |  | Sig. (2-tailed) | .468 |
|  |  | N | 10 |
