# Supplementary material for: Assessment of ChatGPT-generated medical Arabic responses for patients with metabolic dysfunction–associated steatotic liver disease
Source: PLoS One. 2025 Feb 3;20(2):e0317929. doi: 10.1371/journal.pone.0317929 (PMC11790096; doi:10.1371/journal.pone.0317929)
Supplement: S10 Table — (DOCX) [file pone.0317929.s010.docx]

**S10 Table. Arabic questionnaire**

| الأسئلة | **#** |
| --- | --- |
| تم تشخيصي بالكبد الدهني، هل يجب تحويلي إلى أخصائي كبد؟ وهل يجب عمل فحوصات دم أو أشعة؟ | **1** |
| كم يجب علي أن أخسر من وزني عند الإصابة بالكبد الدهني؟ | **2** |
| ما الحمية التي يجب أن أتبعها إذا كنت مصابا بالكبد الدهني؟ | **3** |
| ما الأطعمة التي يجب تجنبها إذا كنت مصابا بالكبد الدهني؟ | **4** |
| هل يمكنني شراب الكحول إذا كنت مصابا بالكبد الدهني؟ | **5** |
| ما الأطعمة التي تؤثر بشكل إيجابي على مرض الكبد الدهني؟ | **6** |
| هل يمكنني استعمال الأعشاب إذا كنت مصابا بالكبد الدهني؟ | **7** |
| هل يمكنني شرب القهوة إذا كنت مصابا بالكبد الدهني؟ | **8** |
| هل يمكنني التدخين إذا كنت مصابا بالكبد الدهني؟ | **9** |
| ما مدة التمارين المناسبة كمصاب بالكبد الدهني؟ | **10** |
| ما التمارين المفضلة للمصاب بالكبد الدهني؟ | **11** |
| هل يجب عمل تمارين الكارديو أو رفع الأثقال إذا كنت مصابا بالكبد الدهني؟ | **12** |
| ما الأدوية التي يجب تناولها عند الإصابة بمرض الكبد الدهني؟ | **13** |
| كيف يمكن المعرفة كمصاب بالكبد الدهني أن حالتي تزداد سوءا؟ | **14** |
| كيف يمكن المعرفة كمصاب بالكبد الدهني أن حالتي تتحسن؟ | **15** |
