# Supplementary material for: Assessment of ChatGPT-generated medical Arabic responses for patients with metabolic dysfunction–associated steatotic liver disease
Source: PLoS One. 2025 Feb 3;20(2):e0317929. doi: 10.1371/journal.pone.0317929 (PMC11790096; doi:10.1371/journal.pone.0317929)
Supplement: S11 Table — (DOCX) [file pone.0317929.s011.docx]

**S11 Table. ChatGPT Generated Responses**

The following ChatGPT interaction history was automatically generated and archived, and can be accessed at:

<https://chat.openai.com/share/b9957e9c-b98b-4980-b376-943b16d3fa15>

In the event that the original link becomes inaccessible, the textual content is provided in the below table for reference and reproducibility:

| **#** | **Input** | **Out put** |
| --- | --- | --- |
| 1 | تم تشخيصي بالكبد الدهني، هل يجب تحويلي إلى أخصائي كبد؟ وهل يجب عمل فحوصات دم أو أشعة؟ | أنا لست طبيبًا، لكني سأحاول تقديم المعلومات العامة حول الكبد الدهني.  إذا تم تشخيصك بالكبد الدهني (Non-Alcoholic Fatty Liver Disease - NAFLD)، فإنه من الجيد أن تستشير طبيبك للحصول على تقييم دقيق لحالتك وتوجيهك إلى العناية اللازمة. قد تحتاج إلى زيارة أخصائي الكبد (أخصائي هضمية أو أخصائي كبد) لتقديم تقييم مخصص وخطة علاج مناسبة.  يعتمد ما إذا كان يجب عليك عمل فحوصات دم أو أشعة على حالتك الصحية الشخصية والتقدير السريري للطبيب. عادةً ما يتم طلب فحوصات دم لتقدير مستويات الإنزيمات الكبدية والدهون في الدم وأداء وظائف الكبد. قد يطلب الطبيب أيضًا إجراء أشعة تصويرية مثل التصوير بالموجات فوق الصوتية أو الموجات المغناطيسية النووية (MRI) لتقييم حالة الكبد بشكل أفضل.  من المهم أيضًا العمل على تغيير نمط حياتك للحد من تطور الكبد الدهني. ذلك قد يشمل تغيير نظامك الغذائي وزيادة ممارسة النشاط البدني، وتجنب استهلاك الكحول، ومراقبة وزنك، ومعالجة حالات مرافقة مثل ارتفاع ضغط الدم وارتفاع مستويات السكر في الدم إذا كانت موجودة.  التوجيه الطبي الشخصي من قبل طبيبك سيكون الأفضل لمعالجة حالتك بشكل فعال. لذا، يجب عليك مراجعة طبيبك لمناقشة خيارات العلاج والاستشارة حول الفحوصات والإجراءات اللازمة بناءً على حالتك الفردية. |
| 2 | كم يجب علي أن أخسر من وزني عند الإصابة بالكبد الدهني؟ | إذا كنت تعاني من الكبد الدهني، فقد يكون فقدان الوزن من الأمور المهمة للتحسين في حالتك. تختلف الاحتياجات الفردية من شخص لآخر، ولا يمكن تحديد الوزن الذي يجب عليك خسارته بدقة دون معرفة تفاصيل حالتك الصحية الكاملة ومستوى تلف الكبد وعوامل أخرى. ومع ذلك، عادةً ما يتم توجيه الأشخاص الذين يعانون من الكبد الدهني إلى تحقيق الأهداف التالية:   1. تخفيف الوزن: إذا كنت تعاني من زيادة في الوزن أو السمنة، فإن فقدان الوزن يمكن أن يكون له تأثير إيجابي على الكبد الدهني. حتى فقدان وزن يبلغ 5-10% من وزن الجسم الإجمالي قد يكون مفيداً. 2. اتباع نظام غذائي صحي: ينصح بتناول نظام غذائي متوازن وغني بالفواكه والخضروات والحبوب الكاملة، وتقليل استهلاك الدهون المشبعة والسكريات المضافة والكحول. 3. ممارسة النشاط البدني: القيام بالنشاط البدني بانتظام يمكن أن يساعد في تحسين حالة الكبد الدهني والسيطرة على الوزن. 4. متابعة طبية: من المهم مراجعة أخصائي الكبد أو الطبيب الخاص بك لتقييم تقدم حالتك وضبط العلاج والتوجيه بناءً على احتياجاتك الفردية.   يجب دائمًا استشارة الطبيب أو أخصائي التغذية لوضع خطة ملائمة لفقدان الوزن تناسب حالتك الصحية الشخصية واحتياجاتك. لاحظ أن فقدان الوزن يجب أن يتم ببطء وبطرق صحية، ولا يجب أن يتسبب في فقدان وزن زائد بسرعة. |
| 3 | ما الحمية التي يجب أن أتبعها إذا كنت مصابا بالكبد الدهني؟ | إذا كنت مصابًا بالكبد الدهني (Non-Alcoholic Fatty Liver Disease - NAFLD)، فإن اتباع حمية صحية يمكن أن يكون له تأثير إيجابي على حالتك. هنا بعض الإرشادات العامة للحمية التي يمكن أن تساعد في إدارة الكبد الدهني:   1. تناول الفواكه والخضروات: تناول كميات كبيرة من الفواكه والخضروات يمكن أن يساعد في توفير الألياف والعناصر الغذائية الضرورية لصحة الكبد. 2. تناول الحبوب الكاملة: يفضل استبدال الحبوب المكررة بالحبوب الكاملة مثل الشوفان والأرز البني والخبز الكامل. 3. تقليل الدهون المشبعة والكوليسترول: تجنب الدهون المشبعة والكوليسترول العالي، وذلك عن طريق تقليل استهلاك اللحوم الحمراء والمنتجات الألبانية الدهنية والأطعمة المصنعة التي تحتوي على دهون مشبعة. 4. الحد من السكريات المضافة: تجنب الأطعمة والمشروبات التي تحتوي على سكريات مضافة بكميات كبيرة، مثل المشروبات الغازية والحلويات. 5. الحد من الأطعمة المعالجة: تجنب الأطعمة المعالجة والأطعمة المقلية والوجبات السريعة، والتي تحتوي عادةً على كميات عالية من الدهون الضارة. 6. الحفاظ على الوزن الصحي: إذا كنت تعاني من زيادة الوزن أو السمنة، فإن فقدان الوزن قد يساعد في تحسين حالة الكبد الدهني. تحدث مع أخصائي تغذية أو طبيبك لتحديد وزن هدف مناسب وخطة للوصول إليه. 7. تقليل استهلاك الكحول: إذا كنت تعاني من NAFLD، يجب عليك تقليل أو تجنب استهلاك الكحول تمامًا. 8. شرب الكثير من الماء: تأكد من شرب كميات كافية من الماء للمساعدة في تطهير الجسم وتحسين وظائف الكبد.   من المهم أن تتحدث مع أخصائي تغذية أو طبيبك لتحديد الحمية الأنسب لحالتك الصحية الفردية ومتابعة تقدمك وضبط الحمية حسب الحاجة. الأهم هو أن تتبع حمية صحية ومتوازنة تدعم صحة الكبد والجسم بشكل عام. |
| 4 | ما الأطعمة التي يجب تجنبها إذا كنت مصابا بالكبد الدهني؟ | إذا كنت مصابًا بالكبد الدهني (NAFLD)، فهناك بعض الأطعمة والمشروبات التي يفضل تجنبها أو تقليل استهلاكها للمساعدة في تحسين حالتك والوقاية من تفاقم المرض. إليك بعض الأطعمة والعوامل التي يجب تجنبها أو تقليل استهلاكها:   1. الدهون المشبعة: تجنب الأطعمة الغنية بالدهون المشبعة مثل اللحوم الدهنية ومنتجات الألبان الكاملة والزبدة. هذه الدهون يمكن أن تسبب تراكم الدهون في الكبد. 2. الدهون المهدرجة والزيوت المهدرجة: تجنب الزيوت المهدرجة والدهون المهدرجة الموجودة في الأطعمة المصنعة والمأكولات السريعة. تلك الدهون يمكن أن تكون ضارة للكبد. 3. السكريات المضافة: حاول تقليل استهلاك السكريات المضافة والمشروبات الغازية والحلويات والحبوب المصنعة التي تحتوي على سكر مضاف. 4. المشروبات الكحولية: يجب تجنب استهلاك الكحول تمامًا إذا كنت تعاني من NAFLD. الكحول يمكن أن يزيد من تلف الكبد وتفاقم الحالة. 5. الوجبات الجاهزة والأطعمة المعالجة: تجنب الأطعمة المصنعة والأطعمة الجاهزة التي تحتوي على كميات عالية من الدهون المهدرجة والسكريات والأملاح. 6. الكربوهيدرات البسيطة: تقليل استهلاك الكربوهيدرات البسيطة مثل الأطعمة المصنعة من الدقيق الأبيض والأرز الأبيض، واستبدالها بالكربوهيدرات الكاملة مثل الشوفان والأرز البني. 7. الأطعمة الغنية بالملح: قلل استهلاك الأطعمة الغنية بالملح، حيث يمكن أن يزيد الصوديوم من ارتفاع ضغط الدم وتفاقم تضرر الكبد. 8. المشروبات الغازية والمشروبات السكرية: تجنب المشروبات الغازية والعصائر المعبأة التي تحتوي على سكر مضاف.   تذكر أن هذه التوجيهات تعتمد على حالتك الصحية الفردية وقد يحتاج تنظيم الحمية الخاص بك إلى تقدير شخصي من قبل أخصائي تغذية أو طبيب. تغيير نمط حياتك الغذائي والالتزام بحمية صحية يمكن أن يساعد في تحسين وضع الكبد الدهني والحفاظ على صحتك العامة. |
| 5 | هل يمكنني شراب الكحول إذا كنت مصابا بالكبد الدهني؟ | عندما تعاني من الكبد الدهني (NAFLD) أو أي مشكلة في الكبد، فإنه يفضل تجنب شرب الكحول بشكل كامل. الكحول يمكن أن يكون ضارًا للكبد ويزيد من خطر تفاقم حالة الكبد الدهني.  شرب الكحول يمكن أن يسبب التلف في خلايا الكبد ويزيد من احتمال تطور التليف الكبدي وتصاعد المشاكل الصحية المرتبطة بالكبد. بالإضافة إلى ذلك، الكحول يمكن أن يزيد من احتمال التهاب الكبد ويجعل من الصعب على الكبد أداء وظائفه بشكل صحيح.  لذا، إذا كنت مصابًا بالكبد الدهني أو لديك أي مشكلة في الكبد، من الأفضل تجنب شرب الكحول تمامًا. إذا كنت تعاني من تحديات في الامتناع عن شرب الكحول، يمكنك التحدث مع طبيبك أو محترف الصحة النفسية للحصول على دعم ومساعدة في التعامل مع هذه القضية. |
| 6 | ما الأطعمة التي تؤثر بشكل إيجابي على مرض الكبد الدهني؟ | هناك العديد من الأطعمة التي يمكن أن تؤثر بشكل إيجابي على حالة الكبد الدهني (NAFLD) وتساعد في تحسين وظائف الكبد وتقليل تراكم الدهون فيه. إليك بعض الأطعمة والمكونات الغذائية التي يمكن أن تكون مفيدة:   1. الأسماك الدهنية: تناول أسماك غنية بأحماض أوميغا-3 مثل السلمون والتونة والسردين، حيث تساعد هذه الأحماض على تقليل الالتهابات وتحسين صحة الكبد. 2. الفواكه والخضروات: تناول الفواكه والخضروات الغنية بالألياف مثل التفاح والبطاطس الحلوة والسبانخ، حيث تساعد الألياف في تحسين صحة الكبد والتحكم في مستويات السكر في الدم. 3. الحبوب الكاملة: استبدال الحبوب المكررة بالحبوب الكاملة مثل الشوفان والأرز البني يمكن أن يكون مفيدًا للكبد. 4. اللبن منخفض الدسم: تناول منتجات الألبان منخفضة الدسم مثل الزبادي الخالي من الدهون يمكن أن يكون أفضل من تناول الألبان العادية غنية الدهون. 5. الزيوت الصحية: استخدم زيوت صحية مثل زيت الزيتون وزيت الكانولا بدلاً من الزيوت المهدرجة. 6. المكملات الغذائية: قد تكون بعض المكملات مفيدة، مثل مكملات فيتامين E وفيتامين D، ولكن يجب استشارة طبيبك قبل تناول أي مكمل غذائي. 7. شرب الكثير من الماء: تناول كميات كافية من الماء يمكن أن يساعد في تطهير الكبد وتحسين وظائفه. 8. الشاي الأخضر: يُعتقد أن الشاي الأخضر يحتوي على مضادات الأكسدة التي يمكن أن تكون مفيدة للكبد.   من الهام جداً الالتزام بنمط حياة صحي وتوازن غذائي متنوع، وتجنب الأطعمة المصنعة والمشروبات الضارة. قبل تغيير حميتك أو تناول مكملات غذائية، يفضل دائمًا استشارة طبيبك أو أخصائي تغذية لضمان أن الخيارات تناسب حالتك الصحية الفردية. |
| 7 | هل يمكنني استعمال الأعشاب إذا كنت مصابا بالكبد الدهني؟ | استخدام الأعشاب كجزء من نمط حياتك الصحي بشكل عام قد يكون مفيدًا، وبعض الأعشاب يمكن أن تكون لها فوائد محددة للكبد. ومع ذلك، يجب أن تكون حذرًا عند استخدام الأعشاب والمكملات العشبية إذا كنت تعاني من مشكلات في الكبد مثل الكبد الدهني (NAFLD)، ويجب عليك النظر في النصائح التالية:   1. استشارة الطبيب: قبل استخدام أي نوع من الأعشاب أو المكملات العشبية، يجب عليك استشارة طبيبك أو أخصائي الكبد. هذا يهم بشكل خاص إذا كنت تتناول أدوية أخرى أو إذا كان لديك مشكلات صحية أخرى. 2. تجنب الجرعات الزائدة: تجنب تناول الجرعات الزائدة من الأعشاب أو المكملات العشبية، حيث يمكن أن يكون لها آثار جانبية غير مرغوب فيها. 3. اختيار الأعشاب بحذر: اختر الأعشاب التي تعرفها جيدًا والتي تعتقد أنها آمنة للاستخدام. بعض الأعشاب مثل الزنجبيل والكركم يمكن أن تكون مفيدة للكبد. 4. تجنب الأعشاب المثيرة للكبد: تجنب الأعشاب التي يُعتقد أنها مثيرة للكبد وقد تكون ضارة بالنسبة لمرضى الكبد. 5. المتابعة الدورية: يجب متابعة حالتك بانتظام من قبل طبيبك وإخباره بأي تغييرات في نمط حياتك الغذائي أو استخدام الأعشاب.   من المهم فهم أن الأعشاب والمكملات العشبية ليست بديلاً للعلاج الطبي الرئيسي والتوجيهات الطبية. يجب أن تُستخدم كجزء من نهج شامل للعناية بصحة الكبد بالتعاون مع الطبيب المختص. |
| 8 | هل يمكنني شرب القهوة إذا كنت مصابا بالكبد الدهني؟ | نعم، يُمكن شرب القهوة بشكل عام إذا كنت مصابًا بالكبد الدهني (NAFLD). بالوجه العام، يُعتبر شرب القهوة آمنًا وقد يكون له بعض الفوائد لصحة الكبد. ولكن هناك بعض النقاط التي يجب مراعاتها:   1. التحكم في السكر والدهون: إذا كنت تعتزم شرب القهوة مع الكبد الدهني، حاول تجنب إضافة السكر والكريمات عالية الدهون إلى القهوة. السكر المكثف والدهون المشبعة يمكن أن تكون ضارة للكبد. 2. الحد من الكميات: تناول القهوة بشكل معتدل. الكميات المعتدلة عادة تعني 3-4 أكواب قهوة يوميًا، ولكن يمكن أن تختلف الاحتياجات من شخص لآخر. 3. تجنب القهوة المضافة: تجنب القهوة المضافة بالسكريات والمواد الكيميائية الصناعية مثل القهوة المثلجة المحلىة والقهوة السريعة التحضير. 4. الانتباه للأعراض الجانبية: بعض الأشخاص قد يكونون حساسين للقهوة وقد يعانون من أعراض جانبية مثل القلق أو الارتجاع المعدي المريئي. في حالة ظهور أي أعراض جانبية، يمكن أن تكون القهوة غير مناسبة لك. 5. استشر طبيبك: إذا كنت قلقًا بشأن تأثير القهوة على حالتك الصحية الخاصة بالكبد الدهني، يجب أن تستشير طبيبك أو أخصائي الكبد. طبيبك يمكنه تقديم نصائح وتوجيهات مخصصة بناءً على حالتك الفردية.   في المجمل، يمكن شرب القهوة بشكل معتدل كجزء من نمط حياتك الصحي إذا كنت مصابًا بالكبد الدهني. ولكن يجب أن تكون حذرًا وتتبع توجيهات الطبيب إذا كانت هناك أية مخاوف خاصة بصحتك الشخصية. |
| 9 | هل يمكنني التدخين إذا كنت مصابا بالكبد الدهني؟ | التدخين غير مفيد لأي شخص، وخاصةً إذا كنت تعاني من مشاكل في الكبد مثل الكبد الدهني (NAFLD)، فإنه يفضل بشدة تجنب التدخين بشكل كامل. التدخين له تأثير سلبي كبير على الكبد وصحتك العامة.  بالإضافة إلى تأثيرات التدخين الضارة على القلب والرئتين والأوعية الدموية، يمكن أن يزيد التدخين من تطور التهاب الكبد وتلفه. قد يزيد التدخين من خطر تطور مشاكل في الكبد ويصعب على الكبد أداء وظائفه بشكل صحيح.  إذا كنت تدخن وتعاني من الكبد الدهني أو أي مشكلة في الكبد، يُفضل بشدة البدء في عملية إيقاف التدخين. يمكنك استشارة طبيبك أو محترف صحة نفسية للمساعدة في وقف التدخين وتطبيق استراتيجيات للتحكم في الإدمان على التدخين. التوقف عن التدخين سيكون خطوة مهمة نحو تحسين صحة الكبد وصحتك العامة. |
| 10 | ما مدة التمارين المناسبة كمصاب بالكبد الدهني؟ | مدة التمارين المناسبة للأشخاص الذين يعانون من الكبد الدهني يمكن أن تتباين من شخص لآخر وتعتمد على مستوى اللياقة البدنية الحالي والقدرة على التحمل. الهدف الرئيسي هو الحفاظ على نشاط بدني منتظم ومناسب يمكن الالتزام به بشكل دائم. إليك بعض الإرشادات العامة:   1. البداية ببطء: إذا كنت غير معتاد على ممارسة الرياضة، فبدأ بتمارين خفيفة وزيادة التدريجي للمستوى الأعلى من النشاط البدني. 2. الهدف: حاول الوصول إلى ما يقل عن 150 دقيقة من النشاط البدني المعتدل في الأسبوع، أو 75 دقيقة من النشاط البدني الشديد أسبوعياً، أو مزيج من الاثنين. وهذه هي التوصيات الصحية للبالغين من قبل منظمات الصحة العالمية. 3. التنوع: حاول ممارسة مجموعة متنوعة من التمارين بما في ذلك المشي، والسباحة، وركوب الدراجات، والتمارين الرياضية، والتمارين بالأوزان. 4. استمع إلى جسمك: انصت إلى جسمك واعتن بأي علامات تشير إلى تعب أو إجهاد زائد. من المهم السماح للجسم بالاستراحة عند الحاجة. 5. استشارة الطبيب: قبل بدء أي برنامج تمارين جديد، خاصة إذا كنت تعاني من مشكلات صحية مثل الكبد الدهني، يفضل استشارة طبيبك للحصول على توجيهات ونصائح خاصة بحالتك الصحية.   من المهم أن تجد النوعية والمدة والشدة التي تناسبك وتجعل التمارين جزءًا من نمط حياتك اليومي. النشاط البدني المنتظم يمكن أن يساعد في تحسين صحة الكبد والعامة واللياقة البدنية، ولكن يجب أن تكون معتدلة ومتوازنة ومستدامة. |
| 11 | ما التمارين المفضلة للمصاب بالكبد الدهني؟ | للأشخاص الذين يعانون من الكبد الدهني (NAFLD)، يمكن الاستفادة من مجموعة متنوعة من التمارين البدنية. هذه التمارين يمكن أن تساعد في تحسين صحة الكبد واللياقة البدنية بشكل عام. إليك بعض التمارين المفضلة للأشخاص الذين يعانون من الكبد الدهني:   1. المشي: المشي هو تمرين بسيط ومتاح للجميع. يمكنك المشي في الهواء الطلق أو داخل المنزل باستخدام مشاية. حاول السير لمدة 30 دقيقة على الأقل في اليوم على مدى أيام الأسبوع. 2. السباحة: السباحة هي تمرين رائع للكبد الدهني، حيث تعمل على تنشيط جميع عضلات الجسم وتعزز اللياقة البدنية. 3. ركوب الدراجة: ركوب الدراجة يمكن أن يكون تمرينًا ممتعًا وفعالًا لتحسين اللياقة البدنية وصحة الكبد. 4. التمارين الرياضية: تمارين القوة مثل رفع الأثقال أو استخدام المقاومة مفيدة أيضًا. تساعد في بناء العضلات وزيادة معدل الأيض. 5. اليوغا: اليوغا تساعد على تحسين المرونة والتوتر العضلي والاسترخاء العام. 6. تمارين الاستطالة: تمارين الاستطالة تساعد على تحسين المرونة وتقليل الإجهاد. 7. تمارين التنفس: تمارين التنفس العميق يمكن أن تساعد في تحسين وظائف الرئتين وزيادة الأكسجين في الجسم.   مهم جدًا أن تبدأ بتمارين بسيطة وتزيد تدريجيًا شدتها ومدتها. يُفضل استشارة طبيبك أو محترف رياضي قبل بدء أي برنامج تمريني جديد، خاصة إذا كان لديك مشاكل صحية أو قلق بشأن مستوى اللياقة البدنية الحالي. الهدف هو الحفاظ على نشاط بدني منتظم ومستدام لتحسين صحة الكبد والعامة. |
| 12 | هل يجب عمل تمارين الكارديو أو رفع الأثقال إذا كنت مصابا بالكبد الدهني؟ | إذا كنت مصابًا بالكبد الدهني (NAFLD)، فإن مزج التمارين الكارديو (التمارين الهوائية) مع تمارين رفع الأثقال (التمارين التي تزيد من القوة) يمكن أن يكون مفيدًا لصحتك العامة وصحة الكبد. الجمع بين الاثنين يمكن أن يقدم فوائد متعددة، لكن يجب أن يتم ذلك بحذر وبناءً على مستوى لياقتك البدنية وحالتك الصحية الفردية. إليك بعض الأمور التي يجب مراعاتها:   1. التمارين الكارديو:    - العمل على القلب والأوعية الدموية وزيادة حرق السعرات الحرارية.    - مناسبة لتحسين استجابة الجسم للأنسولين والتحكم في مستويات السكر في الدم.    - تشمل التمارين الكارديو المشي، والجري، وركوب الدراجة، والسباحة، وغيرها. 2. تمارين رفع الأثقال:    - تساعد في بناء العضلات وزيادة القوة العامة.    - يمكن أن تساعد في تحسين مستوى الطاقة والقدرة على أداء الأنشطة اليومية بشكل أفضل.    - يجب أن تكون مدروسة بعناية لتجنب إصابات العضلات والمفاصل.   مهم جدًا أن تبدأ بتمارين بسيطة وتزيد تدريجيًا من الشدة والمدة. يفضل استشارة طبيبك أو محترف رياضي قبل بدء أي برنامج تمريني جديد، خاصة إذا كنت تعاني من مشاكل صحية أو قلق بشأن مستوى لياقتك البدنية الحالي. الهدف هو الحفاظ على نشاط بدني منتظم ومتوازن لتحسين صحة الكبد والعامة. |
| 13 | ما الأدوية التي يجب تناولها عند الإصابة بمرض الكبد الدهني؟ | إدارة مرض الكبد الدهني (NAFLD) تعتمد على حالتك الصحية الفردية وشدة المرض. في العديد من الحالات، لا تكون هناك حاجة إلى الأدوية خصوصًا إذا تم التعرف على المشكلة في وقت مبكر وتم اتباع نمط حياة صحي يتضمن الغذاء السليم والنشاط البدني.  ومع ذلك، في حالات متقدمة من NAFLD أو إذا كان هناك مشاكل صحية أخرى مرتبطة بالكبد، قد يقرر الطبيب وصف الأدوية. بعض الأدوية والمكملات يمكن أن تكون مفيدة في بعض الحالات وتشمل:   1. الأدوية لمعالجة مقاومة الأنسولين: بعض الأدوية مثل ميتفورمين (Metformin) يمكن أن تساعد في تحسين استجابة الجسم للأنسولين وتحسين مستويات السكر في الدم. 2. الأدوية لخفض الكوليستيرول: الأدوية مثل الستاتينات يمكن أن تستخدم لخفض مستويات الكوليستيرول في الدم إذا كان ذلك مطلوبًا. 3. المكملات الغذائية: بعض المكملات مثل فيتامين E أو أوميغا-3 قد تكون مفيدة في بعض الحالات، ولكن يجب استشارة الطبيب قبل تناول أي مكمل غذائي.   يجب على الأشخاص الذين يعانون من NAFLD العمل بشكل وثيق مع فريق طبي أو أخصائيين في الكبد لتقييم حالتهم واتخاذ القرارات المناسبة بشأن العلاج والأدوية. العلاج الفعال لمرض الكبد الدهني يعتمد بشكل كبير على النمط الحياتي والتغذية والنشاط البدني. |
| 14 | كيف يمكن المعرفة كمصاب بالكبد الدهني أن حالتي تزداد سوءا؟ | يمكن مراقبة حالة الكبد الدهني (NAFLD) وتقييم تطورها من خلال عدة عوامل وعلامات. إليك بعض الطرق التي يمكنك استخدامها للتعرف على تدهور حالتك:   1. فحوصات دمية: يمكن لاختبارات الدم تقديم مؤشرات مهمة عن صحة الكبد. تتضمن هذه الاختبارات مستويات أنزيمات الكبد مثل AST وALT ومستوى بروتين الكبد ومستويات الأنزيمات الكبدية الأخرى. ارتفاع هذه المؤشرات قد يشير إلى تدهور حالة الكبد. 2. السونار أو التصوير بالرنين المغناطيسي: يمكن استخدام الفحوصات التصويرية مثل السونار أو التصوير بالرنين المغناطيسي لتقدير حجم وتكوين الكبد والكشف عن أي تغييرات تحدث في الدهون أو النسيج الكبدي. 3. بيوبسي: في حالة الشك الكبير بوجود تدهور في حالة الكبد، قد يقوم الطبيب بإجراء بيوبسي لأخذ عينة من النسيج الكبدي لتقدير مدى التلف والالتهاب في الكبد. هذا الاختبار يمكن أن يقدم معلومات دقيقة حول حالة الكبد. 4. أعراض جسدية: يمكن للأعراض مثل الألم في الجزء العلوي من البطن أو الشعور بالغثيان أو ارتفاع في ضغط الدم أو تغيرات في الوزن تشير إلى تدهور في حالة الكبد. 5. متابعة النمط الحياتي: إذا لاحظت زيادة في استهلاك الكحول، أو تغييرات كبيرة في نمط الغذاء أو نقص النشاط البدني، فهذه العوامل يمكن أن تساهم في تدهور حالة الكبد.   مهم جدًا أن تستشير الطبيب بشكل دوري وتتبع توجيهاته لمراقبة حالة الكبد وتقييم تطورها. من المهم أن تتبع نمط حياة صحي، بما في ذلك الأكل الصحي والنشاط البدني المنتظم، للحفاظ على صحة الكبد والحد من تدهور الحالة. |
| 15 | كيف يمكن المعرفة كمصاب بالكبد الدهني أن حالتي تتحسن؟ | لمعرفة ما إذا كانت حالة الكبد الدهني (NAFLD) تتحسن، يمكنك اتخاذ الإجراءات التالية:   1. فحوصات دمية: تتضمن فحوصات الدم قياس مستويات أنزيمات الكبد مثل AST وALT والبروتينات الكبدية الأخرى. إذا كانت هذه القيم تتحسن وتعود إلى المعدلات الطبيعية، فهذا قد يشير إلى تحسن في حالة الكبد. 2. التصوير الشعاعي: يمكن استخدام السونار أو التصوير بالرنين المغناطيسي لمراقبة تغييرات في هيكل الكبد. إذا كانت الدهون في الكبد تقل أو تصبح أقل بروزًا على الصور، فهذا قد يشير إلى تحسن. 3. متابعة الأعراض: إذا كنت تعاني من أعراض مثل الألم في البطن أو الغثيان أو الضيق في التنفس ولاحظت تحسنًا في هذه الأعراض، فهذا قد يشير أيضًا إلى تحسن في حالة الكبد. 4. النمط الحياتي: إذا كنت قد قمت بتحسين نمط حياتك بشكل عام من خلال تغييرات في الغذاء وزيادة النشاط البدني والحد من تعاطي الكحول والتخلص من السمنة، فهذا يمكن أن يساهم في تحسن حالة الكبد. 5. متابعة الطبيب: من المهم متابعة طبيبك بشكل منتظم لتقييم حالة الكبد ومراقبة تحسنها. الطبيب سيكون قادرًا على توجيهك بناءً على الاختبارات والفحوصات وإعطاء توجيهات أفضل لمتابعة الحالة.   مهم جدًا الالتزام بتوصيات الطبيب والمحافظة على نمط حياة صحي ومستدام للحفاظ على تحسن حالة الكبد. تذكر أن تجديد تقييم حالتك ومراقبتها بشكل منتظم هو جزء مهم من الرعاية الصحية والوقاية من تدهور حالة الكبد. |
